# Review of Polymers and Nanocomposites Based on Hyaluronic Acid and Zinc Oxide Nanoparticles in Synthesizing Microneedles for Transdermal Drug Delivery

**DOI:** 10.3390/polym18020217

**Published:** 2026-01-14

**Authors:** Kolawole S. Dada, Roman O. Olekhnovich, Falia F. Zaripova

**Affiliations:** Chemical Engineering Center, ITMO University, Saint Petersburg 191002, Russia; r.o.olekhnovich@mail.com (R.O.O.); faliabio@yandex.ru (F.F.Z.)

**Keywords:** transdermal patch, hyaluronic acid, zinc oxide nanoparticles, drug delivery, microneedle

## Abstract

The objective of this review is to provide an in-depth overview of the current state of research on microneedles for drug delivery, with emphasis on composite systems, particularly those based on hyaluronic acid (HA) and zinc oxide as composite systems. The review discusses the advantages, challenges, and future directions associated with the use of these materials for microneedle-based drug delivery. Hyaluronic acid, which is naturally found in human tissue, has been shown to enhance drug permeation and improve skin hydration, while ZnO has provided high mechanical durability and prevented microbial and bacterial growth. The use of dissolvable microneedles is a viable substitute for traditional drug delivery methods such as oral or intravenous administration. Ongoing research is being conducted to further improve the performance, safety, and effectiveness of microneedles for various drug delivery applications.

## 1. Introduction

### 1.1. Background of Study

Transdermal drug delivery to a targeted area, where it is required for healing, is a very advantageous and quite a novel methodology in modern pharmaceutical and pharmacological applications.

**Figure 1:**
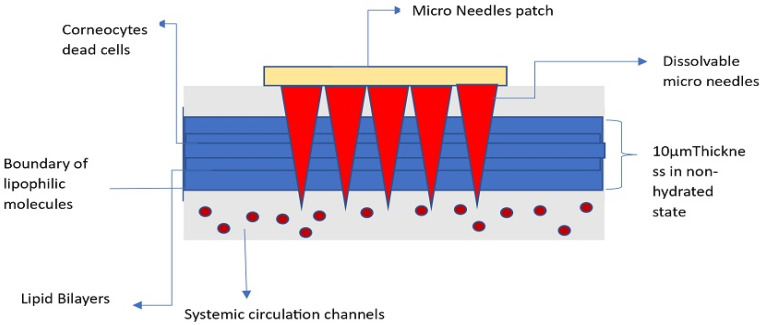
Pictorial view of the dissolvable microneedle drug’s release into the systemic layer.

This method of drug delivery offers several benefits over other conventional methods, such as oral and parenteral routes. It is non-invasive, with improved patient compliance, which helps to reduce the dosage intake frequency [1]. Another advantage of microneedles is that they provide targeted drug delivery to specific areas of the body, mitigating the risk of systemic side effects. Microneedles can also be designed to release drugs at a controlled rate, providing sustained delivery over an extended period. These properties are very advantageous for long-term drug administration. Microneedles with a length of 500 µm and above can penetrate the stratum corneum of the skin or other biological tissues for painless and non-invasive drug administration [2]. Immediately when microneedles are applied to the skin, they puncture the stratum corneum, relatively known as the outermost layer of the skin [3]. This layer is made up of dead skin cells and acts as a barrier to prevent the entry of foreign substances into the body. Microneedles can penetrate the stratum corneum by creating tiny channels in the skin, through which drugs or vaccines are administered into the underlying tissues, as demonstrated in Figure 1. The penetration of microneedles into the skin is typically painless and does not inflict substantial harm to the skin or underlying tissues. Microneedles have been recognized as a promising drug delivery system that can overcome the limitations of traditional drug delivery methods. Microneedle technology can offer regulated release of medications, leading to controlled levels of drugs circulating in the blood, preventing the ups and downs that are frequently associated with taking drugs orally [4]. The depth of penetration can be adjusted depending on the desired target tissue. The tissue gradually absorbs the drugs released from the needle. However, the mechanical properties, biocompatibility, and drug release characteristics influence their ability to deliver drugs. In some cases, additional biodegradable polymers such as polyvinyl alcohol (PVA), polyvinylpyrrolidone (PVP), or chitosan may be blended with HA to modify the HA.

In establishing the relevance of the study, researchers have developed microneedles made from a composite material of silk fibroin and hydroxyapatite, resulting in increased mechanical strength and enhanced drug release properties. In the case of microneedle fabrication, composite materials can provide improved mechanical strength, flexibility, and drug loading capacity. There are other carbon-based nanomaterials that are being investigated for microneedle fabrication that include graphene oxide, carbon nanotubes, and metal–organic frameworks. These materials present the possibility for improved biocompatibility, electrical conductivity, and controlled drug release.

### 1.2. Problem Statement and Research Gap

There are so many stimulating questions and research opportunities in the use of biocompatible and functional materials, which make hyaluronic acid and zinc oxide nanoparticles still underexplored. Most of the research in the past five years has focused on using HA and ZnO with additional components, which limits the effects of just these two credible materials. As a result, there is a need to investigate the HA-ZnO composite for microneedle synthesis to score its inherent properties and therapeutic potentials. Another major gap lies in the study of penetration, which solely relies on ultrasound activation or general performance evaluation [5]; the work is done without quantifying critical insertion parameters or measuring tip sharpness, mechanical strength, or the insertion force threshold. In some research, only a few reports have been recorded regarding the structural integrity of microneedles during application, after usage, or during storage for a long time. This restriction hinders the possibility of reproducibility.

Studying the storage effect of the microneedle requires more emphasis on temperature and humidity effects. Most merely assume the stability of HA-ZnO under different environmental conditions. The arising question will focus on how other variables, like chemical activities and light exposure, affect the physical structure of the microneedle based on the activity of ZnO ROS (reactive oxygen species) generation during storage. HA, being a very hydrophilic polymer, will require analyzing its hygroscopic effects on long-term storage.

Moreover, ZnO has confidently shown its anti-inflammatory and antimicrobial properties, its drug release kinetics characteristics, and its biocompatibility in other research. Researchers have not fully explored the potential of its properties as a microneedle. The kinetics of ZnO nanoparticles when released from the HA matrix, their cytotoxicity at varying concentrations, and their interaction with the skin microbiota are questions still unanswered.

Finally, the molecular weight of hyaluronic acid influences its viscosity, glass transition temperature, degradation, and cellular interaction. This molecular weight is rarely compared in existing studies. The decision to use low- and-high-molecular-weight HA can significantly dictate the microneedle’s mechanical properties, dissolution rate, and immune response in all cases. The combination of these two (LMW and HMW) to create a balance in the epidermis and dermis during drug administration is yet to be explored. This represents a promising opportunity to optimize the performance function.

### 1.3. Objectives of the Review

The objective of the review is centered and focused on addressing various fabrication techniques by examining the potential of using hyaluronic acid and zinc oxide nanoparticle composites in microneedle synthesis. The review aims to carry out the following:

1. Analyze the influence of composite ratios of HA-ZnO and their effect on mechanical properties, penetration efficiency, and drug release functionality;
2. Comparison of various fabrication strategies and their potential for drug delivery;
3. Identify design and output limitations as a guide for the future design of HA-ZnO microneedle devices.

## 2. Literature Review

### 2.1. Overview of Microneedle Technology

#### Classification of Microneedles

**Figure 2:**
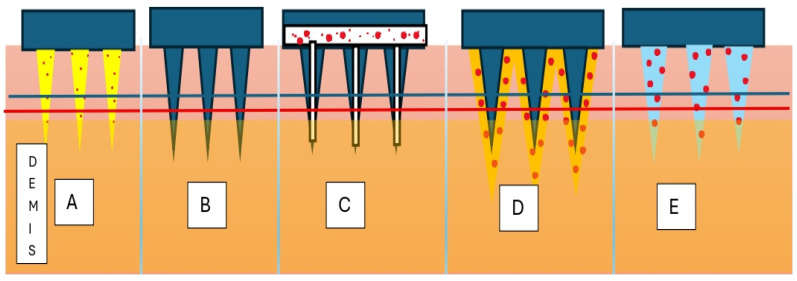
Various types of microneedles. (**A**) Hydrogel microneedle, (**B**) solid microneedle, (**C**) hollow microneedle, (**D**) coated microneedle, and (**E**) dissolving microneedle.

**Figure 3:**
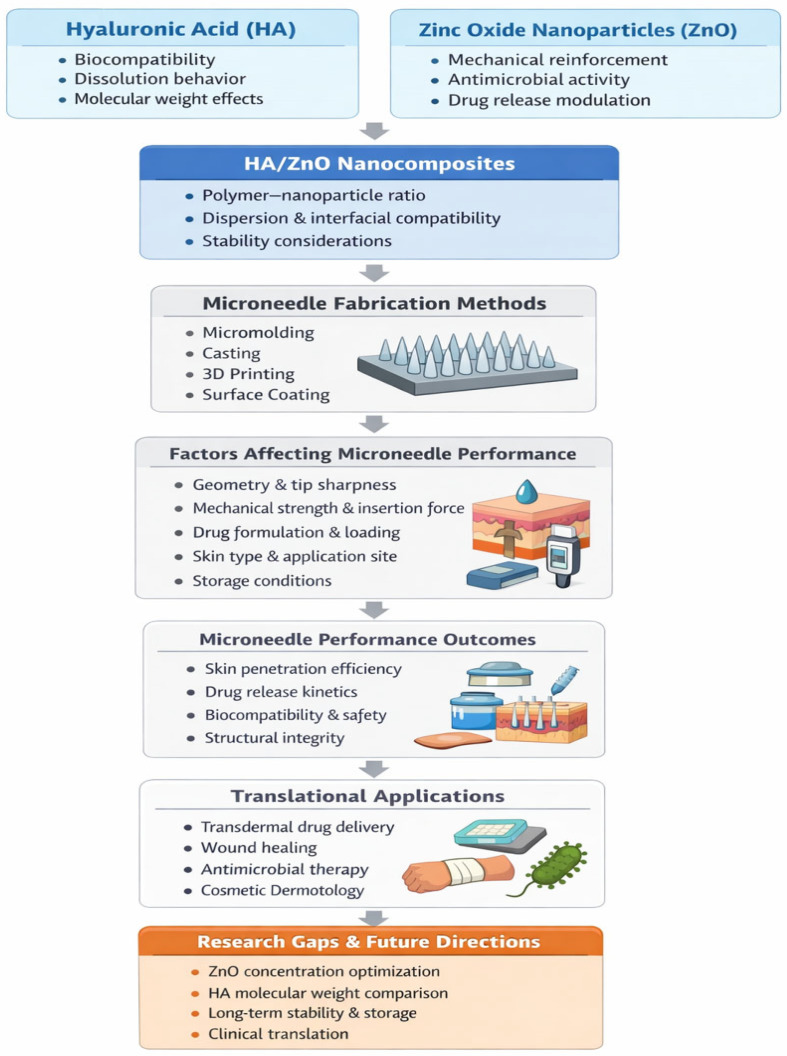
Schematic overview of the framework of the review summary. This illustrates relationships between HA/ZnO nanoparticles, microneedle fabrication methodology, orientation of performance, translational application, and identified research gaps.

Microneedles for drug delivery can be described, as shown in Figure 2, as hydrogel-forming, solid, hollow, coated, or dissolving and are used to target specific layers of the skin or deliver drugs into the bloodstream [6,7,8]. However, microneedles also have some limitations. The manufacturing of these microneedles requires specialized equipment, and their usage may be limited by the size and type of drug being delivered. They may also be expensive compared to traditional injection methods. The needles are designed to be sharp enough to penetrate the skin but not so sharp as to cause pain or injury. In the case of solid microneedles, the drug or vaccine is coated onto the surface of the needles, and as the needles penetrate the skin, the drug is released into the underlying tissues. In the case of hollow microneedles, the drug or vaccine is injected directly into the subcutaneous tissue through the hollow channel of the needle. Lastly, dissolving microneedles are made from biodegradable materials that dissolve in the skin over time. As the microneedles dissolve, the drug or vaccine is released into the surrounding tissue. An overview of the conceptual design, guiding this review is demonstrated in Figure 3.

Table 1 displays the category of microneedles. Solid microneedles are made of a single piece of material, usually stainless steel or polymer, and are coated with the drug formulation. There are several coating methods approved for channeling drugs, which are dip coating, casting, and deposition [9,10]. Hollow microneedles have a small channel running through the center of the needle, allowing the drug to be injected directly into the tissue. A recent review discussed the introduction of hollow microneedles for introducing mesenchymal stem cells, keratinocytes, and artificial scaffolds of decellularized extracellular matrix (dECM) to support the proliferation and integration of cells for tissue regeneration [11]. Coated microneedles are also less invasive than traditional hypodermic needles, requiring less injection volume and reducing the risk of tissue damage. Additionally, coated microneedles have the potential to be self-administered. Patients can apply for their medication at home, reducing the need for frequent visits to the doctor’s office. The application of coated microneedles has been shown to effectively facilitate the skin penetration of various macromolecules such as bovine serum albumin, interferon-alpha, desmopressin, parathyroid hormone, insulin, peptide A, recombinant human erythropoietin alfa, antisense oligonucleotides, erythropoietin, ovalbumin, bovine pancreatic ribonuclease A, and human growth hormone, etc. [12,13]. On the other hand, dissolving microneedles is a novel drug delivery system that uses tiny needles to deliver therapeutic agents directly into the body. These microneedles are made of biocompatible materials that dissolve upon contact with the skin and release therapeutic agents into the surrounding tissue. Earlier microneedles were typically made from biodegradable materials such as sugar or gelatin. The therapeutic agents are loaded into microneedles in a variety of ways, including coating the needles with a solution containing the drug or embedding the drug directly into the needles [14]. Hydrogel-forming microneedles are a recent advancement in the field of drug delivery. They are composed of biocompatible materials that can form a gel-like structure upon hydration, allowing for the controlled release of therapeutic agents into the body. These microneedles are typically made of a polymer matrix that is embedded with the desired drug or vaccine [15]. Once the needle is inserted into the skin, the moisture present in the tissue causes the polymer matrix to swell and form a hydrogel, effectively trapping the drug inside [16,17]. Hydrogel-forming microneedles offer the ability to control the release of drugs, allowing for sustained delivery over an extended period. This feature is particularly advantageous in the case of vaccines, as a single dose can provide long-lasting immunity.

However, microneedles also have some limitations. One of the major disadvantages is that they are not suitable for delivering large volumes of drugs, as the needle size is tiny. Additionally, the drug loading capacity of microneedles is also limited, which may be a drawback in cases where high drug doses are required. Furthermore, the cost of manufacturing microneedles is currently higher compared to traditional drug delivery methods, which could limit their widespread adoption. Microneedle technology is relatively new and still requires further research and development to fully explore its potential.

### 2.2. Methodology of Fabrication of Microneedles

Fabrication methods of microneedles keep evolving and improving based on the materials used, cost, defining precision, and scalability. The fabrication method directly influences the geometry, performance, mechanical strength, and drug loading capacity of the microneedles. The fabrication techniques can be categorized into molding-based, additive manufacturing, photolithographic/microfabrication, lithography, metallic processing, and coating or dissolving methods. Each method mentioned has its distinct advantages and disadvantages as described and summarized in Table 2.

#### 2.2.1. Molding-Based Fabrication

Under this section, we have micro-molding and solvent casting as one of the most established methods for producing dissolving microneedles. The procedure is to cast the polymer solvent and nanoparticle blend in a substrate or microcavity, which are made from PDMS or silicone. A process of centrifugation or degassing follows, then drying and demolding.

#### 2.2.2. Additive Manufacturing

This procedure utilizes the direct digital fabrication of microneedles with precision centered on geometry and dimensions. The techniques employed, such as stereolithography (SLA), digital light processing (DLP), and two-photon polymerization (2PP), allow for a layer-by-layer structure in computer-aided designs. The result provides high flexibility for customizable hollow microneedle structures, which can be integrated with pharmaceutical ingredients. This method has its limitations, such as the availability of bioprinting materials, and it also restricts scalability.

#### 2.2.3. Drawing Lithography

This method uses precise pulling parameters to stretch viscous polymer droplets into conical microneedles. While it cools, it solidifies and retains the tips and shapes. It is low cost and does not require specific and expensive mold equipment. The control over the uniformity of the needles’ geometry and length posed a problem.

#### 2.2.4. Photolithography and MEMS Methods

The microelectromechanical system (MEMS) was one of the earliest methods used in silicon microneedle fabrication. This procedure uses photoresist patterning and reactive ion etching to design microneedles to scale. There are predictable, precise geometry fabrications, which include hollow and beveled types of microneedles, which are suitable for drug delivery. It is costly and limited to usage and design for non-biodegradable materials.

#### 2.2.5. Metallic and Coating Techniques

In this microneedle fabrication category, laser micromachining, electroplating, or the photochemical etching of metal foils is used. The synthesized microneedle produces excellent quality in terms of mechanical strength and can be used repeatedly. Drugs can be attached to microneedles using dip coating, spray coating, or inkjet printing, which enables rapid drug release upon painless insertion.

### 2.3. Materials for Microneedles

#### 2.3.1. HA in Drug Delivery

Hyaluronic acid is a naturally occurring polysaccharide that is found in various tissues of the body, including the skin, joints, and eyes. It has a unique ability to bind and retain water, which makes it an effective moisturizer and lubricant. HA is also biocompatible, biodegradable, and non-immunogenic, making it an attractive excipient for drug delivery applications. HA-based drug delivery systems have been investigated for various routes of administration, including oral, through injections, and on the skin. The use of HA in drug delivery can enhance drug bioavailability, target specific tissues, and prolong drug release. Additionally, HA can also act as a scaffold for tissue engineering applications [18].

#### 2.3.2. Advantages of Using Hyaluronic Acid in Dissolving Microneedles

- Biocompatibility: Hyaluronic acid is a biocompatible material that is naturally present in the body, making it safe for use in medical applications;
- Biodegradability: Hyaluronic acid is biodegradable, which means it can be broken down and metabolized by the body over time. This property reduces the risk of long-term toxicity and enables the development of safer and more effective drug delivery systems;
- Enhances drug delivery: Hyaluronic acid can enhance drug delivery by increasing the permeability of the skin and facilitating the diffusion of drugs through the skin. It can also improve drug solubility and stability, resulting in increased drug loading and prolonged drug release;
- Reduction in pain and discomfort: The use of hyaluronic acid in dissolving microneedles can reduce pain and discomfort during drug delivery due to its lubricating and cushioning properties;
- Wound healing and tissue regeneration: Hyaluronic acid has been shown to promote wound healing and tissue regeneration, making it an ideal material for use in dissolving microneedles for medical applications such as wound healing and tissue repair.

**Figure 4:**
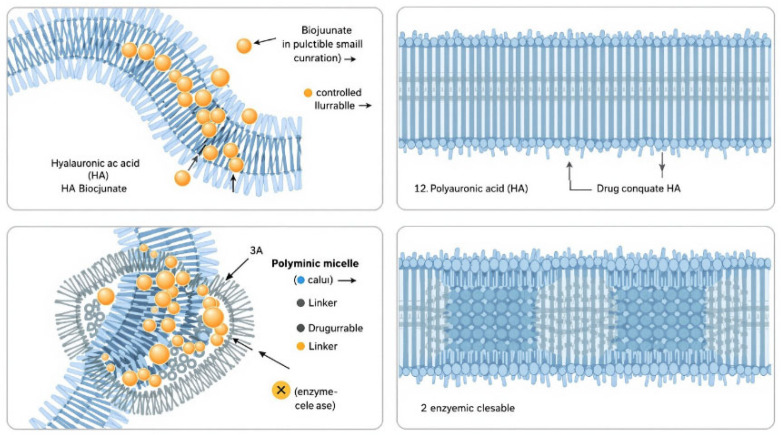
Drug delivery formulations using HA and its derivatives.

A study conducted by [19,20] investigated the use of HA in dissolvable microneedles (DMNs) for the delivery of complex substances used for improving facial wrinkles in different areas of the skin. A schematic illustration of Hyaluronic acid based drug delivery formulation using HA and its derivatives is illustrated in Figure 4. Researchers found that HA-based DMNs were able to deliver tranexamic acid (TXA) effectively and efficiently, with a higher drug concentration in the skin compared to traditional topical applications. They also noted that HA-based DMNs were able to dissolve quickly, leaving no residue in the skin. Another study conducted by [21] investigated the use of HA in DMNs for the delivery of insulin. The researchers found that HA-based DMNs were able to effectively deliver insulin into the bloodstream of diabetic rats, reducing blood glucose levels for up to hours after administration. Overall, the use of hyaluronic acid in DMNs has shown promising results for drug delivery applications. Several studies have reported the successful use of HA in drug delivery applications for various therapeutic agents, including anticancer drugs, anti-inflammatory agents, and antibiotics. The versatility of HA in drug delivery is due to its ability to form various types of drug delivery systems, such as nanoparticles, hydrogels, and microparticles [22,23]. Overall, the use of HA for drug delivery holds significant promise in the field of drug delivery and tissue engineering. However, further research is needed to optimize HA-based drug delivery systems for clinical use. Hyaluronic acid-based drug delivery systems have shown promise in cancer therapy due to their ability to target specific cells and tissues [22]. Hyaluronic acid has been shown to target cancer cells due to its ability to bind to CD receptors that are overexpressed in many types of cancer cells [24].

#### 2.3.3. Influence of HA Molecular Weight and Chemical Modification

Another study approach, which is titled “In Vitro Biodegradability of Phosphorylated Hyaluronic Acid by Testicular Hyaluronidase,” investigated the biodegradation of phosphorylated hyaluronic acid. Although there were no exact numerical values regarding the rate of degradation, it further discusses the role of testicular hyaluronidase in the degradation of phosphorylated HA. This finding signifies that the biodegradability of HA can be supported by chemical modifications through enzymatic activity, which could be supported directly for controlled release applications in drug delivery systems [25].

Moreover, there is other research with the title “Hyaluronic Acid: The Influence of Molecular Weight on Structural, Physical, Physicochemical, and Degradable Properties of Biopolymer,” which delves deeply into the methods and influence of the molecular weight of HA and how it affects its structural, physical, physicochemical, and degradable properties. This study also lacks evidence of the degradation rate; the research specifies the importance of molecular weight in deciding the overall characteristics of HA. Higher molecular weights usually correlate with better and improved viscoelasticity and higher resistance to degradation, which can be advantageous in certain biomedical application fields but may be disadvantageous for utility in other areas where rapid clearance is desired [26]. In another published article titled “A multifunctional anti-inflammatory drug that can specifically target activated macrophages, massively deplete intracellular H_2_O_2_, and produce large amounts of CO for a highly efficient treatment of osteoarthritis,” the study demonstrates that hyaluronic acid-based nanocomposites can function actively in ROS-rich inflammatory microenvironments, which enables immune cell interactions and targets oxidative control. This highlight is a fact about the functional role of modified HA integration with nanoparticles for wound healing and transdermal therapy [27].

#### 2.3.4. The Addition of ZnO as a Filler to Enhance Drug Delivery

Zinc oxide (ZnO) nanoparticles have gained attention as a potential nanoparticle for drug delivery, thanks to their distinctive characteristics, such as biocompatibility, toxicity control, and large surface area-to-volume ratio [28]. Recent research has focused extensively on the application of ZnO nanoparticles for drug delivery purposes. The significant surface area-to-volume ratio of ZnO nanoparticles allows them to carry a considerable number of drugs that can be gradually released over time. The rate of drug release can be managed by altering the size and surface properties of these nanoparticles. Besides their drug delivery capabilities, ZnO nanoparticles have demonstrated antibacterial, anti-cancer [29], antioxidant, anti-inflammatory, and antifungal properties, making them suitable for wound healing applications [30]. Nevertheless, it is crucial to mention that the toxicity of ZnO nanoparticles remains a topic of discussion, and further research is required to comprehensively understand their safety profile.

Furthermore, some research described in the provided source details the production of a green synthesis of a hyaluronan/zinc oxide (HA/ZnO) nanocomposite used as a potential cancer treatment medication. The preparation process involves certain conditions, which include the use of hyaluronic acid (HA) and zinc oxide (ZnO) with specific measured concentrations of 1.0% *w*/*v* by dissolving HA in sodium hydroxide (1.0% *w*/*v*) with some adequate stirring for 1 h and leading to the formation of a nanocomposite with unique properties. In the experimental research, seaweed (*Sargassum muticum*) was suspended in 100 mL of distilled water in a 200 mL Erlenmeyer flask, which was heated to 100 °C, and then filtered through a Whatman 41 filter paper to obtain the seaweed extract. A 1 mM aqueous solution of zinc acetate dehydrate was mixed with 50 mL of the seaweed water extract, which was then added to the HA solution under constant stirring for 2–3 h at 70 °C. Finally, the final mixture was centrifuged at 200× *g* for 8 min, which was followed by washing with distilled water and drying for 4 h at 100 °C. The final product, the HA/ZnO nanocomposite, was stored in airtight bottles at room temperature until use [31].

Moreover, the stability of ZnO nanoparticles under physiological conditions needs additional investigation to guarantee their efficacy in a drug delivery system.

#### 2.3.5. Factors Affecting the Performance of HA-ZnO-Based Dissolving Microneedles

The performance of HA-ZnO DMNs is a function of several factors:

- Hyaluronic acid concentration: The concentration of hyaluronic acid in the microneedles can affect their mechanical properties, such as strength and stiffness, which limit their ability to penetrate the skin and deliver drugs;
- Microneedle size and shape: The size and shape of the microneedles can also influence their mechanical properties and drug delivery efficiency. Smaller microneedles are generally better at penetrating the skin but may have a lower drug loading capacity;
- Drug properties: The physicochemical properties of the drug being delivered can also impact microneedle performance. Factors such as solubility, molecular weight, and charge can affect drug loading, release, and diffusion through the skin;
- Manufacturing process: The method used to manufacture microneedles can affect their physical and mechanical properties, such as size, shape, and strength;
- Formulation additives: The addition of other formulation additives, such as penetration enhancers or stabilizers, can also affect the performance of microneedles and drug delivery. Overall, the performance of dissolving microneedles made using hyaluronic acid is influenced by several factors, and the optimization of these factors is critical for achieving the desired drug delivery efficiency and therapeutic effect;
- Particle size: The size of ZnO particles can significantly impact the dissolution rate and mechanical strength of DMNs. Smaller particles typically dissolve more quickly but may result in a lower mechanical strength. Conversely, larger particles can provide better mechanical strength but may dissolve more slowly;
- Concentration of ZnO: The concentration of ZnO in the DMN formulation can also impact its dissolution rate and mechanical strength. Higher concentrations of ZnO can result in faster dissolution but may lead to decreased mechanical strength;
- Type and concentration of excipients: Excipients are added to DMN formulations to improve their mechanical properties. The type and concentration of excipients used can affect the dissolution rate and mechanical properties of DMNs;
- Storage conditions: The storage conditions of DMNs, such as temperature and humidity, can affect their stability and performance. Improper storage conditions can result in changes to the mechanical strength and dissolution rate of DMNs.

#### 2.3.6. Advantages of Using Hyaluronic Acid and ZnO in Microneedles

However, some general performance features of HA and ZnO-based DMNs are as follows. Hyaluronic acid-based MNs: These DMNs are known for their low insertion force, minimal pain sensation during application, and high drug delivery effectiveness. However, they have a relatively low drug loading capacity and exhibit a moderate rate of drug release. They are biodegradable, compatible with a wide range of drugs, and biocompatible. Nonetheless, their manufacturing costs can be expensive. ZnO-based DMNs: These DMNs have moderate to high drug delivery effectiveness and a high drug loading capacity. Their high insertion force makes them appropriate for delivering drugs to deeper skin layers. They are biocompatible, low cost, and compatible with a wide range of drugs. They exhibit a moderate to high rate of drug release, and their biodegradability is moderate. Additionally, they do not cause skin irritation. More details of the comparison of HA and ZnO can be seen in Table 3.

### 2.4. Functional Role of Zinc Oxide (ZnO) Nanoparticles in HA-Based Dissolving Microneedles

ZnO plays dynamic roles in the hyaluronic acid (HA)-based dissolvable microneedle synthesis. The properties of ZnO extend it beyond a reinforcer to mechanical property functional integration, anti-microbial support, and drug kinetic release aid. The integration of zinc oxide nanoparticles makes it viable for transdermal drug delivery.

#### 2.4.1. Mechanical Reinforcement and Structural Stability

Zinc oxide can act as a reinforcer for improving stress distribution and load transfer during insertion. The uniform dispersion of zinc oxide nanoparticles in the polymer matrix makes improvements in the fracture resistance and uniform compression force load across the array. It also reduces localized deformation under the axial load by resisting polymer chain movement. Several ZnO composites have demonstrated an improved young’s modulus with optimized nanoparticle loading. However, excessive zinc oxide loading can cause agglomeration or cluster formation, which weakens the mechanical integrity and affects the functionality of the microneedle [32].

#### 2.4.2. Influence of ZnO on Dissolution and Drug Release Behavior

The addition of nanofillers such as ZnO in the polymer matrix of HA affects the dissolution kinetics of HA-based DMNs. ZnO can alter polymer–water interaction as a water uptake modifier. This behavior introduces microporosity in HA. ZnO can promote localized channels for hydration, which enables faster drug release into the body during microneedle administration. Conversely, a higher concentration of zinc oxide can reduce the dissolution rate by increasing the density matrix through the formation of a polymer–particle network that slows down polymer erosion. It is important to have a balance in the size of zinc oxide particles to control the dissolution pathways suitable for drug release [33].

#### 2.4.3. Antimicrobial, Anti-Inflammatory, and Wound Healing

The significance of adding zinc oxide to the polymer matrix relates to its inherent antimicrobial and bioactive functions. ZnO exhibits a broad spectrum of antimicrobial properties against both Gram-positive and Gram-negative bacteria, through a process of reactive oxygen species (ROS) generation. This property is advantageous and useful where microchanneling is required for drug delivery to act as a barrier of infection on the skin. Zinc oxide has demonstrated antioxidant and anti-inflammatory activity, which contributes to improved wound healing. On the other hand, HA provides a hydrated and bioactive environment that supports cell proliferation for tissue repair. The interaction between these two materials, HA and ZnO, is good for wound healing, drug delivery, and cosmetic applications [34].

#### 2.4.4. Interfacial Interaction Between HA and ZnO Nanoparticles

The performance of the web matrix of HA-ZnO composite microneedles is influenced by the interfacial interactions between the polymer and nanoparticles. These are defined by electrostatic interaction, hydrogen bonding, and coordinating interaction. These can influence nanoparticle dispersion and adhesion. Surface modification methods such as chelation, PEGylation, and pH modification have been shown to support the ZnO interaction with HA, which leads to no phase separation, reduced particle aggregation, and improved mechanical consistency across the needle array [35].

#### 2.4.5. Safety, Toxicity, and Regulatory Considerations

Despite the enumerated advantages of interaction and output, the use of ZnO nanoparticles in microneedles requires taking safety and toxicity into account. ZnO toxicity is influenced by particle size, concentration, surface chemistry, and its exposure to ROS generation. A prolonged exposure to nanoparticles may increase the cytotoxicity or inflammatory response. HA-based microneedles offer a safety advantage by enabling controlled ZnO delivery at the skin surface, thereby reducing systemic exposure. Irrespective of this assertion, comprehensive in vivo and in vitro assessments are required to study the long-term skin compatibility [36].

### 2.5. Composite Materials for Microneedle Fabrication

Composite materials are being increasingly explored for the fabrication of microneedles due to their unique properties and potential benefits. A composite material is a combination of two or more materials that have different physical and chemical properties, resulting in a material with enhanced properties compared to the individual components. A summary of polymer-nanoparticle materials that can be used for potential microneedle fabrication, based on functional roles and other possible advantages can be seen in Table 4.

Research that focuses on composite materials made of hyaluronic acid and zinc oxide nanoparticles for microneedle syntheses is scarce. There are several related studies that discussed the composite function of behaviors in skin, wound, acne, and antibacterial contexts. The properties of these composites expand into penetration, inflammation, and storage period. These numerous studies on HA and ZnO nanoparticles are summarized below in Table 5.

Table 5 displays a comparison analysis of existing HA-ZnO composites and their biological functionality; however, most research does not illustrate the microneedles’ objectives and only relies on external functionalization material for the activation of drug release, like composite films. Some research omits the quantitative assessment of needle penetration, mechanical integrity, and performance after storage. The recurring gaps in the isolation of the HA-ZnO composite limit its translational relevance.

### 2.6. Factors Affecting Microneedle Performance

Microneedle performances are measured based on several key parameters that support their functionality for drug delivery. The essence of minimally invasive technology is to be able to outperform the conventional methods of drug administration without limitations. This section analyzes the design strategies to overcome the limitations and improve efficacy. A comprehensive summary of various factors governing microneedle performance for drug delivery is provided in Table 6.

#### 2.6.1. Needle Geometry

**Figure 5:**
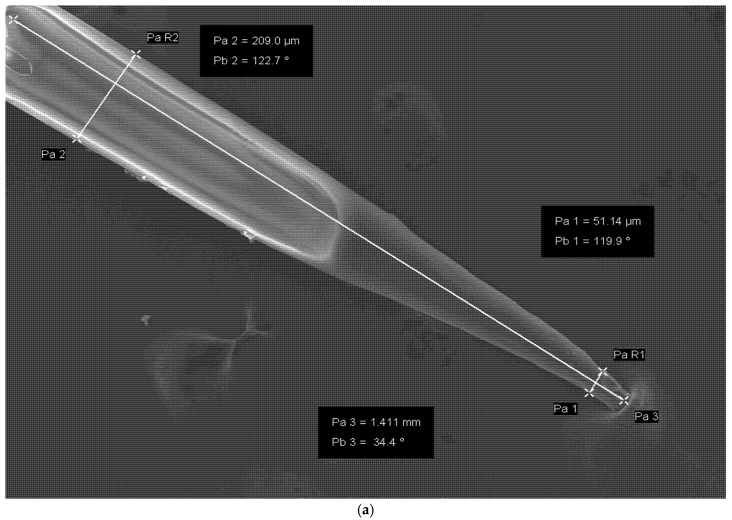

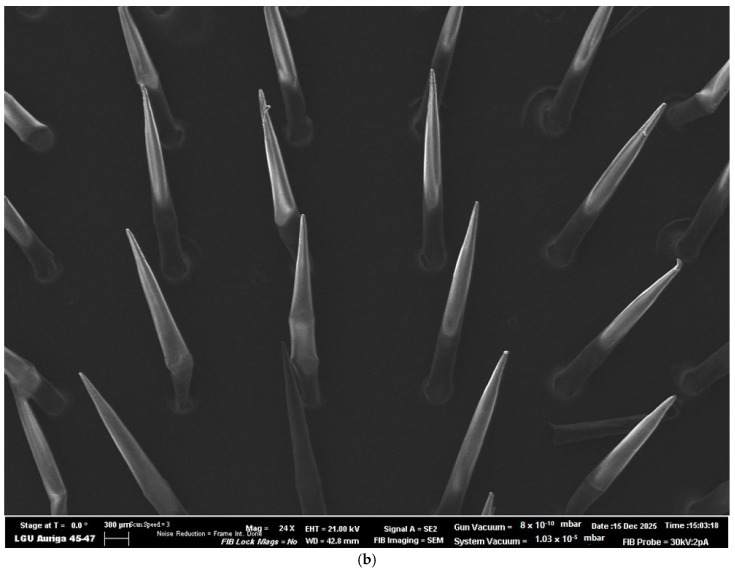
Representative SEM image of HA-based dissolving microneedles, illustrating the key geometric dimensions such as needle height, tip sharpness, and base width. These features are critical determinants of insertion efficiency and mechanical stability, as discussed. The image is included for illustrative purposes to support discussion of microneedle geometry in HA-based systems. Where image (**a**) represents a single microneedle from the arrays, and (**b**) depicts arrays of microneedles.

The needle geometry is the physical structure of the design that depicts its length, angular proportions, thickness, tips, array arrangement, width, and every other dimension that contributes to the structural integrity of the microneedle. The needle geometry is built on a balance of penetrating force, compression reaction, and drug kinetics as can be seen in Figure 5 using SEM image analysis. There are different needle lengths within the range of 150–3000 µm, tip sharpness of 1–25 µm, and base width of 50–250 µm [48].

#### 2.6.2. Drug Formulation

Drug formulation is the primary required strategy in designing microneedles, as the whole essence of the research is drug delivery. Strategic questions for absolute value placement include drug solubility, required concentration, kinetic release rate, and drug particle size. How can we encapsulate these properties into the geometry of the microneedle for efficacy?

#### 2.6.3. Composite Material Ratio

The synthesized microneedle, is made solely from hyaluronic acid to study the mechanical strength and reaction of compression force on the tips before comparing it to a reinforced microneedle array using ZnO nanocomposites. The composite ratio for performance will be designed to suit the mechanical strength, drug release kinetics, and dosage control.

#### 2.6.4. Skin Type and Conditions

The skin is the primary barrier for drug infusion through microneedles; people have different skin types, which range from dry skin to oily skin, sensitive skin, and a combination of both dry and oily skin. Skin types influence skin thickness, elasticity, hydration, barrier integrity, surface morphology, and drug penetration and absorption. This highlights the importance of proposing a specific ratio of HMW and LMW compounds in the synthesis of microneedles to support the epidermis’ hydration before the penetration and infusion of drugs.

#### 2.6.5. Application and Duration

The application interface of the skin varies with different body regions, which is dependent on varying skin thicknesses, vascularization levels, and mechanical properties. Areas with a thicker stratum corneum require longer or stronger applications to maintain the dosage intake compared to areas with thin, well-vascularized surfaces. The longer the diffusion of drugs into the skin lasts, the greater the possibility of skin irritation. Hence, the microneedle design must be optimized to minimize adverse effects.

#### 2.6.6. Manufacturing Method

A labeling strategy must be incorporated for various skin types, with varying thicknesses across the body that must be accounted for, to maximize the drug’s efficacy. Microneedle designs should address the administration surface to promote the drug’s release rate [12].

## 3. Methodology of the Proposed Study

### 3.1. Research Design and Approach

The essence of the research design is to create an analytical procedure to evaluate fabrication methodologies for microneedle synthesis. This research will specifically focus on composites made from hyaluronic acid (HA) and zinc oxide nanoparticles (ZnO NPs). Our research will focus on the comparison and optimization of fabrication parameters that are based on materials used in relation to performance outcomes. This will help define the mechanical strength, dissolution behavior, and interfacial compatibility. We plan to monitor the formation of microstructure analysis and track the possible potential phase separation that may occur.

### 3.2. Proposed Fabrication Process

**Figure 6:**
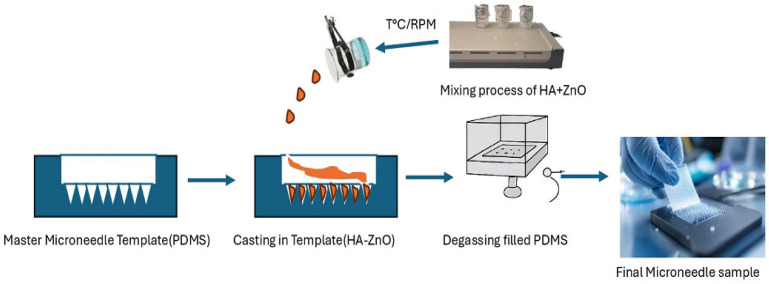
Schematic view of the micro-molding process and demolding of microneedles.

The fabrication method proposed to be used for DMNs is solvent casting micro-molding techniques, and this method has been widely used by other researchers because of its advantages. PDMS is basically used as a mold designed to the specifications of the desired microneedles. The mold is gradually filled with the varying concentrations of the composite, mixed as in Table 7 under the experimental parameters and optimization, in addition to a controlled sample of HA only. The mixture is then placed inside a centrifugal device to ensure that it flows to the mold cavities, and another variant is to degas using a vacuum desiccator at 70–90 kPa. Thereafter, the drying procedure is conducted under various temperature conditions and constant relative humidity. After the drying process, the microneedle is removed carefully from the mold, as seen in Figure 6, and other characterizations are studied. This whole procedure combines the biocompatibility and water solubility of HA and the antimicrobial and mechanical effects of ZnO, based on its purpose for drug delivery.

### 3.3. Experimental Parameters and Optimization

The optimization of the material for microneedle studies based on HA and varying concentrations of ZnO for effective performance and improvement in its properties will follow the designed study pattern, as tabulated below in Table 7 The experimental table will guide the optimization of the absolute values for microneedle selection. Other physical properties studied in this review, like microneedles’ sharp tips, adequate insertion force, and consistent degradation profiles, will help redefine its future usage.

### 3.4. Characterization and Performance Evaluation

Subsequent to optimization research, it is necessary to characterize and assess geometry, mechanical integrity, and functional performance. The morphology analysis can be carried out using an optical microscope or scanning electron microscopy (SEM), which will eventually help to determine sharpness and dimension uniformity. In terms of mechanical analysis, the compression or fracture force will help evaluate the skin penetration force and strength. Other analyses, like dissolution and drug release, will predict the degradation behavior, while antimicrobial assays will confirm the efficacy of the incorporation of ZnO.

## 4. Conclusions and Future Perspectives

### 4.1. Conclusions

The synthesis of microneedles using composite materials from HA-ZnO offers significant promise in the advancement of the biomedical sector. The benefits are mostly from the biocompatible polymer matrices and functional nanomaterials in solving the constraints of limitations, and this projects clinical maturity in terms of applications. There is a need to understand property values, such as mechanics, biostability, molecular interactions, and their therapeutic effects. Future research must also prioritize other variables’ impacts, such as the molecular weights of HA mixing ratios along with the ZnO concentration with respect to the evaluation of other variable parameters, to study the storage longevity, insertion efficiency, antimicrobial effects, and inflammatory responses.

### 4.2. Future Perspectives

The HA-ZnO microneedles have shown feasible and promising results; however, several research gaps remain:

1. Engineered designs and computational modeling

By using computational simulations and the experiment design we can predict the optimal values for the composite ratios in terms of HA’s molecular weight, ZnO’s particle size, surface chemistry, and the geometry of the microneedle. This accounts for interfacial interactions that will enhance the functionality and efficacy of drug release through dissolution control;

2. Surface functionalization

The use of surface modification agents, which include PEGylation, chelation, and cross-linkers, can help to improve the nanoparticle dispersion of ZnO nanoparticles and Zn^2+^ ion release in the polymer matrix. The avoidance of nanoclusters and agglomeration will result in an even distribution of mechanical reinforcement and controlled dissolution. System evaluations are required to study how the modification influences pharmacokinetics and biocompatibility;

3. Scalable fabrication and reproducibility

The development of a standard and reproducible prototype remains a challenge, particularly for HA-ZnO systems where nanoparticle aggregation or sedimentation during mold filling may affect geometry. Options like micro-molding techniques, 3D printing, and automation should be explored to facilitate large-scale production while maintaining uniformity, microneedle morphology, and drug-loading consistency;

4. Performance assessment

Future research should evaluate microneedle performance under conditions such as skin types, inflammatory or irritating responses, and long-term storage, while integrating dissolution kinetics, drug release behavior, antimicrobial efficacy, and Zn^2+^-related safety protocols. Characterization will strengthen the translational potential;

5. Regulatory and clinical considerations

The clinical application of HA-ZnO microneedles requires clear safety, toxicity, and regulatory studies. The long-term impact of ZnO nanoparticles on the skin and systemic circulation are of significant concerns.

Nanotechnology, biomedical engineering, and material science should be integrated for advanced fabrication techniques. This is the key to the future of synthesizing HA-ZnO microneedles. There should be an intentional focus on reproducible fabrication interfaces, mechanical strength, and drug release evaluation.

## Figures and Tables

**Table 1:** Types of microneedles.

Types of Microneedles	Fabrication Technique	Advantages	Disadvantages
Solid microneedles.	Techniques include micro-molding, photolithography, and laser cutting.	It is simple and easy to fabricate, highly reproducible, and exhibits excellent mechanical strength.	It is limited to surface penetration, which restricts its use for some applications.
Hollow microneedles.	This technique requires drawing or etching hollow needles from silicon or metal wafers.	It can be used for various applications, such as transdermal delivery, interstitial fluid sampling, and vaccine delivery.	The fabrication process is complex and expensive; it is also prone to clogging and requires more force to penetrate.
Coated microneedles.	Techniques include dip coating, spray coating, or electrospinning a polymer or drug formulation onto the microneedles.	This is easy to fabricate and can be used for vaccine delivery and interstitial fluid sampling.	The coating process can be uneven and may not fully cover the microneedle, leading to inconsistent drug delivery.
Dissolving microneedles.	Techniques require casting or molding biodegradable polymers loaded with drugs.	It is easy to use, minimizes needle reuse and needlestick injuries, and can be used for various applications, such as vaccine delivery.	Its application is limited to the delivery of low-molecular-weight drugs, and the drug release rate may be affected by various factors.
Hydrogel microneedles	The process involves mixing hydrogel polymers with drugs and casting the mixture into microneedles.	It can deliver a wide range of drugs, including proteins and peptides, and it can be used for various applications, such as vaccine delivery and transdermal drug delivery.	Various factors such as skin hydration and temperature, as well as pH and temperature, can affect the gelation process.

**Table 2:** Comparing the advantages and disadvantages of HA microneedle fabrication techniques.

Materials and Fabrication Techniques	Advantages	Disadvantages
Hyaluronic acid microneedles fabricated via photolithography.	There is precise control over microneedle geometry and size, uniform microneedle distribution, a high aspect ratio of microneedles, and good mechanical strength.	This method uses expensive equipment and processes; it has limited flexibility in changing microneedle shape and size, microneedles may not completely dissolve, and it can be difficult to remove residual photoresist.
Hyaluronic acid microneedles fabricated via drawing lithography.	It is a simple and low-cost technique; it shows flexibility in changing microneedle shape and size, microneedles dissolve quickly, and it possesses good mechanical strength.	There is limited control over microneedle distribution, microneedle height may not be uniform, and microneedles may be damaged during removal from the mold.
Hyaluronic acid microneedles fabricated via micro-molding.	It has high output and low cost, flexibility in changing microneedle shape and size, uniform microneedle distribution, microneedles that dissolve quickly, and good mechanical strength.	The procedure displays limited control over microneedle height; it may require release agents to remove microneedles from the mold, and microneedles may be damaged during removal.
Hyaluronic acid microneedles fabricated via electrospinning.	It has the advantages of a high ratio of microneedles, flexibility in changing microneedle shape and size, good mechanical strength, and the ability to incorporate other materials into microneedles.	It shows low yield and low output; microneedles are poorly distributed, dissolve incompletely, and may require specialized equipment.
Hyaluronic acid microneedles fabricated via laser ablation.	There is an advantage of high precision and control over microneedle shape, size, and excellent mechanical properties.	It has limited control over microneedle distribution; microneedle height may not be uniform, and microneedles may be damaged during removal from the mold.

**Table 3:** Advantages of HA and ZnO as drug carriers.

Advantages	Hyaluronic Acid	ZnO
Biocompatibility	Yes	Yes
Water Solubility	High	Low
Skin Hydration	Yes	No
Drug Delivery	Yes	Yes
Anti-inflammatory	Yes (HMW)	Yes
Non-toxic	Yes	Yes, at a low concentration
Cost-effective	No	Yes

**Table 4:** Composite materials for possible microneedle fabrication.

Biopolymer	Nanoparticle	Advantages	Disadvantages
Chitosan	Silver	It is antimicrobial and biocompatible.	It possesses poor mechanical strength [37].
Chitosan	Gold	It has antimicrobial, biocompatible, tunable plasmonic properties.	It is expensive [38].
Hyaluronan	Silver	It is biocompatible and it promotes wound healing.	It has limited mechanical strength [39].
Gelatin	Methacrylate	It is biocompatible, simple to fabricate, and tunable in its mechanical properties.	It may suffer from swelling and degradation in vivo [40].
Alginate	Iron Oxide	It is biocompatible; it can be used for magnetic targeting.	It has limited mechanical strength [41].
PLA	Carbon Nanotubes	It is biocompatible; it can be used as a sensor, and multifunctional composites are formed.	It has limited mechanical strength [42].
Sodium Alginate	Zinc Oxide	It is biocompatible, antimicrobial, promotes wound healing, and is simple to fabricate.	It exhibits poor mechanical strength [43].

**Table 5:** Summary of the composite film and microneedle polymer composite (HA-ZnO + X).

Materials	Performance	Penetration and Storage Life	Remark	Ref
HA + ZnO composite via biomimetic mineralization.	HA coating improves ZnO stability and mitigates side effects. The HA + ZnO promotes cell proliferation, reduced sebum secretion, anti-inflammatory effects, and prevention of scar formation.It exhibits strong antibacterial activity on Cutibacterium acnes but less effect against the epidermis.	The product is not applicable (optical administration). For storage, it shows a boost in acidic pH.	HA + ZnO composite film is very promising when used as MN.	[44]
Multifunctional HA microneedle patches embedded by cerium/zinc-based composites (ZCO–HA).	Accelerated wound healing without systemic toxicity. High antibacterial resistance, ROS generation, high anti-inflammatory effects via NF-kB signaling, improved angiogenesis, and cell migration.	The product has demonstrated effective penetration in animal models. The storage effect and duration were not accounted for.	Zn^2+^ with other metals can improve anti-inflammatory effects.	[45]
Microneedle patch with ultrasound-responsive ZnO/ZnTCPP nanoparticles.	Reduces inflammatory markers (TNF-α, ILs, MMPs). Promotes fibroblast proliferation for skin repair and generates ROS to kill acne.	It is effective with ultrasound; passive insertion is not quantified. The storage details were not reported.	The detailed information is very relevant to MN synthesis.	[46]
HA/PVA/ZnO membrane (nanocomposite films).	It promotes fibroblast growth and improved wound healing in vivo compared to the control membrane. It also exhibits sustained antibacterial activities.	The film does not apply to the non-MN format. Storage details were not reported.	Film is promising for microneedle synthesis.	[47]

**Table 6:** Factors affecting performance of microneedles for drug delivery.

Factors Affecting Performance	Description
Needle geometry	This accounts for drug delivery efficiency, insertion force, and pain level during application.
Drug formulation	This impacts drug solubility, stability, and concentration, affecting the drug loading capacity, release rate, and effectiveness.
Composite material ratio	This affects themechanical properties, drug release rate, and biocompatibility specifically for materials like hyaluronic acid (HA) and zinc oxide (ZnO).
Skin types and conditions	This strongly influences skin thickness, hydration, and barrier function, impacting drug penetration and absorption.
Application site and duration	This promotes delay in drug delivery efficiency, drug distribution, and potential for skin irritation depending on the location and duration of application.
Manufacturing method	This impacts the mechanical properties, drug release rate, and consistency of drug-loaded microneedles (DMNs).

**Table 7:** Parameters for optimization.

Sample ID	ZnO Content (wt.%)	Crosslinker/Chelating Agent	Stirring Speed (rpm)	Mix. Temp (°C)	Dry. Temp (°C)	Controlled R/H (MgCl_2_)	Drying Time (Days)
S1	0	Citric Acid	300, 800, 1500	20, 40, 60	25, 40, 80	33%	xx
S2	1	Citric Acid	300, 800, 1500	20, 40, 60	25, 40, 80	33%	xx
S3	3	Citric Acid	300, 800, 1500	20, 40, 60	25, 40, 80	33%	xx
S4	5	Citric Acid	300, 800, 1500	20, 40, 60	25, 40, 80	33%	xx

## Data Availability

No new data were created or analyzed in this study.
